# Adaptative Responses of Common and Tartary Buckwheat to Different Altitudes

**DOI:** 10.3390/plants11111439

**Published:** 2022-05-28

**Authors:** Aleksandra Golob, Neja Luzar, Ivan Kreft, Mateja Germ

**Affiliations:** 1 Biotechnical Faculty, University of Ljubljana, Jamnikarjeva ulica 101, 1000 Ljubljana, Slovenia; aleksandra.golob@bf.uni-lj.si (A.G.); neja.luzar2@gmail.com (N.L.); 2 Nutrition Institute, Tržaška 40, 1000 Ljubljana, Slovenia; ivan.kreft@guest.arnes.si

**Keywords:** common buckwheat, Tartary buckwheat, physiological traits, environmental conditions, elevation

## Abstract

Environmental conditions at different elevations are harsher at higher elevations and impose constraints upon plants. The response of common and Tartary buckwheats to environmental conditions at elevations between 300 and 1180 m above sea level (asl) was studied. In common buckwheat, grown at the highest elevation, there was an increased investment in secondary metabolism, and decreased investment in primary metabolism, since the production of UV-absorbing compounds was enhanced while the amounts of chlorophylls and carotenoids decreased. In Tartary buckwheat, the amounts of UV-absorbing compounds, chlorophylls and carotenoids were similar in plants grown at different elevations, indicating better adaptation to conditions at higher elevations. Common and Tartary buckwheat plants from Podbeže had thicker leaves than plants from the two other locations. This could be a response to high radiation in the very sunny position in Podbeže.

## 1. Introduction

Climatic conditions and agroecosystems are strongly interconnected. The distribution and production of crops largely depend upon climatic conditions, which are site-specific and identified mainly as differences in rainfall, light conditions and temperature over the years [1]. Irregular and erratic rainfall, rises in temperature, extreme weather, flooding and prolonged drought in ambient climates are all noticeable events of climate change in nature. These induce changes in plant morphology, physiology, phenology, reproduction, species distribution, community structures, ecosystems and the evolutionary processes of species, all of which can affect yields and the spread of infectious diseases and pests [1]. Conditions at higher elevations can be harsh and may impose different constraints upon plants. Temperatures and precipitation are lower, the growth season is shorter, and snow often persists much longer at higher elevations [2,3]. The intensity of UV-B radiation increases sharply at higher altitudes, while the daily total solar radiation is similar along the elevation gradient [4]. The increase in UV-B radiation (290–320 nm) ranges from between 6 and 8% [4] to 20% [5] per 1000 m of elevation. UV radiation, particularly UV-B radiation, can generate free radicals in plants that damage susceptible tissues and cells [6], impacting agricultural production [7]. Recent results showed that plants can adapt to the environmental changes that accompany high altitudes by decreasing leaf area and by increasing leaf thickness, mesophyll tissue thickness, and stomatal density [8]. Plants originating from high elevations were generally shorter and produced less biomass, while their phenology did not vary consistently [9].

Plants from the *Polygonaceae* family have numerous calcium oxalate (CaOx) druses, which can protect plants against herbivory [10]. Production of CaOx druses is also one of the mechanisms for regulation of total free Ca levels in plant tissues and organs [11]. The light-scattering functions of CaOx crystals contribute to an even dispersion of the light penetrating into shaded leaf tissue [12], enabling leaves to use the incoming light flux more efficiently [13].

Common buckwheat (*Fagopyrum esculentum* Moench) and Tartary buckwheat (*F. tataricum* (L.) Gaertn.) are underutilized crops from the *Polygonaceae* family. Buckwheat has attracted much interest and is becoming one of the most important alternative crops for healthy food [14]. Common buckwheat is generally grown at lower altitudes in Asia and Europe, while Tartary buckwheat is grown at higher altitudes in small areas of marginal and unproductive land in countries in Southeast Asia [15]. It has been reported that cultivation of five common buckwheat variants in the Himalayan region is restricted to elevations between 500 and 2500 m. Above 2500 m, the Tartary buckwheat replaces the common buckwheat. The areas of origin of the wild ancestors of common buckwheat and Tartary buckwheat are thought to be the Chinese provinces of Yunnan and Sichuan [16], and Zhang et al. [17] recently reported that Tartary buckwheat originated in western China. In China and Nepal, Tartary buckwheat grows at higher elevations than common buckwheat [18] and has thus adapted to the harsh environmental parameters of higher altitudes. 

Our research attempted to discover how common and Tartary buckwheat respond at physiological and morphological levels to environmental conditions at different elevations. As Tartary buckwheat originated in higher elevations, we presumed that these plants would be less vulnerable to the environmental constraints that predominate at higher elevations.

## 2. Results and Discussion

### 2.1. Soil Properties, Weather Conditions and Physiological Parameters of Buckwheats

Characteristics of soils and weather conditions at experimental locations are presented in Table 1. Soil from Ljubljana (Lj) was classified as clay loam and had similar characteristics to those of soil from Podbeže (Po), which was classified as loam–clay loam, while soil from Javorje (Ja) was classified as loam–silt loam and had a higher proportion of total carbon and nitrogen, and a higher concentration of potassium, than soil from Ljubljana and Podbeže.

Mean temperatures at the highest location, Javorje, 1180 m above sea level (m asl), in June and July are about 10 °C lower than in Podbeže (600 m asl) and Ljubljana (300 m asl). In Javorje, there is also more rain than in other locations, especially in July.

Despite the differences in weather and soil conditions between locations, both common (CB) and Tartary buckwheat (TB) plants were found to be in good condition at all locations. The relatively high value of the potential photochemical efficiency of photosystem (PS)II, which is close to the theoretical maximum of 0.83, indicates that experimental plants did not suffer irreversible damage to their PSII reaction centers [19]. There were no significant differences between CB and TB in stomatal conductance at different locations, and values were relatively high (more than 300 mmol/m^2^s) (data not shown).

### 2.2. Biochemical Characteristics

**Figure 1:**
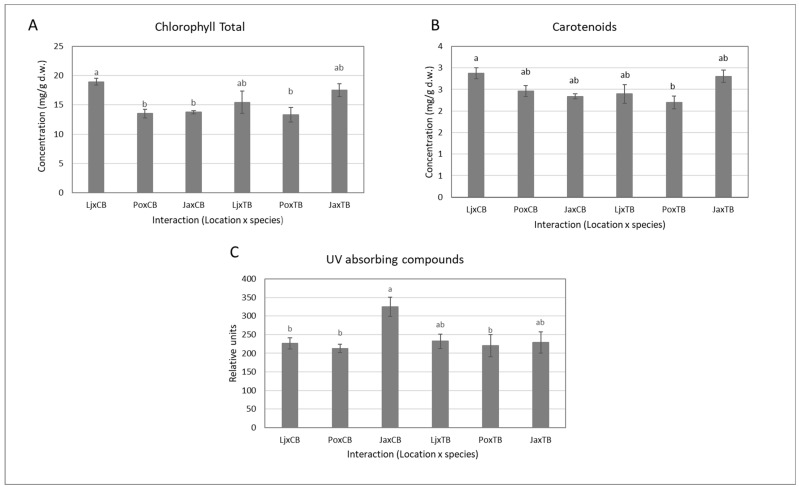
The effect of growth location on the concentrations of chlorophylls (**A**), carotenoids (**B**) and UV-absorbing compounds (**C**) in leaves of CB and TB. Data are means ± standard deviation. Means followed by different letters are significantly different at *p* < 0.05 (n = 8).

Statistical analysis of the results showed that the content of chlorophylls, anthocyanins and UV-absorbing compounds in buckwheat leaves depended on plant growth location but was independent of the species of buckwheat (Table 2). Differences between locations were seen only for common buckwheat (CB). Leaves from CB grown at the highest altitude in Javorje had a significantly lower content of chlorophylls and higher content of UV-absorbing compounds than leaves from plants grown in Ljubljana at the lowest altitudes (Figure 1).

The higher content of chlorophylls in CB plants from the lowest locations suggests that these plants were growing in favorable conditions. The synthesis of chlorophylls demands energy and if plants grow in supportive conditions, they have enough energy to support the primary metabolism. In conditions of stress, the trade-off between primary and secondary metabolism can be directed toward secondary metabolism. UV-absorbing compounds such as flavonoids and related phenylpropanoids are secondary metabolites and the most important compounds that absorb UV radiation [20]. They are located in the upper epidermis and represent the first barrier to sunlight, which can damage vulnerable tissue in plants. The number of UV-absorbing compounds in common buckwheat was highest in plants from the highest elevation for CB, but not for TB (Figure 1). UV radiation increases at higher elevations [4], and with the synthesis of UV-absorbing compounds, plants can protect processes and structures such as DNA from damage caused by UV radiation [21].

In the current study of CB, the total content of UV-absorbing compounds in leaves increased with altitude, while the chlorophyll content showed the opposite trend in St. John’s wort, which was grown in a continuum of habitats from lowlands to highlands [22]. In 2017, Golob et al. [23] found a similar pattern. Wheat plants treated with Se and exposed to ambient UV radiation produced the highest number of UV-absorbing compounds and the lowest levels of photosynthetic pigments. In the current research, we presumed that, at higher elevations, there would be a trade-off between the plant’s investment in primary and secondary metabolisms, as the synthesis of UV-absorbing compounds was enhanced while that of chlorophylls decreased. Tartary buckwheat (TB) plants failed to show the same patterns. There were no differences between the number of chlorophylls and of UV-absorbing compounds in plants from different elevations (Figure 1). It is possible that TB possesses enough UV-absorbing compounds for efficient protection and the plants did not use additional energy in their expanded synthesis. Compared to CB, TB originates from higher elevations. These plants may be better adapted to higher elevation without additional synthesis of protective compounds. It has been reported that the production of UV-absorbing compounds is either dose-dependent or not. Dose independence has been mainly identified in plant colonies in high-radiation environments [24]. Tartary buckwheat grown under different UV-B levels showed that UV-B-absorbing compounds and rutin concentrations became essentially ambient, but this did not occur under elevated levels of UV-B [25]. Suchar and Robberecht (2016) [26] have suggested that this is possibly related to the costs needed to prevent stress due to UV-B at the highest radiation level.

### 2.3. Morphological Characteristics

Plants from the middle elevation (Podbeže) had thicker leaves and palisade tissue than plants from other locations. Of the two species, TB plants had thinner leaves and palisade tissue (Table 3). At the same time, TB did not contain higher levels of chlorophylls and UV-absorbing compounds per dry weight than CB, which means significantly higher levels of chlorophylls and UV-absorbing compounds per leaf area. The better potential of TB to cope with environmental constraints was indicated. Otherwise, thicker leaves allow greater concentrations of chlorophyll per leaf area [27], and this was found in our study. Thinner leaves intercept light better [27]. Plants from Javorje grew in a more shaded environment, and with thinner leaves, they could increase their light interception. CB from Javorje had a higher number of UV-absorbing compounds than CB from other locations, and this prevented the penetration of potentially harmful UV radiation to the sensitive mesophyllic tissue. On the other hand, plants from Podbeže were fully irradiated almost all day and their thicker leaves offered protection against excessive radiation.

**Figure 2:**
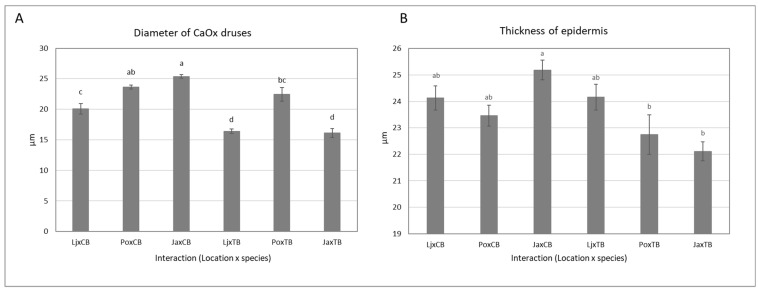
The effect of growth location on concentration diameter of CaOx druses (**A**) and leaf epidermis thickness (**B**) of CB and TB. Data are represented as means ± standard deviation. Means followed by different letters are significantly different at *p* < 0.05 (n = 8).

In both species, the number of CaOx druses was highest in plants from the lowest elevation (Table 2). However, in CB, the dimensions of CaOx druses were smaller in plants grown at the lowest altitude (Figure 2). Because CaOx druses were mainly located along the main veins, this suggests their structural role in supporting leaf tissue. 

Golob et al. [13] revealed an effect of CaOx druses in the optical properties of buckwheat plants. They found a positive correlation between the density of CaOx druse crystals and the light reflectance of the blue, green, yellow and UV-B parts of the light spectrum and a positive correlation between the percentage of the surface area of CaOx druses and leaf transmittance in the green and yellow parts of the light spectrum of Tartary buckwheat leaves. In addition, the number, size and distribution of CaOx druses could additionally contribute to the more efficient use of incoming light.

## 3. Materials and Methods

### 3.1. Experimental Plan

Seeds of common buckwheat (*Fagopyrum esculentum* Moench) (CB) and Tartary buckwheat (*F. tataricum* (L.) Gaertn.) (TB) were sown in sites at different elevations: in Ljubljana (300 m asl; 46.048313 N, 14.473318 E), in Podbeže near Ilirska Bistrica (600 m asl; 45.547141 N, 14.152469 E) and in Javorje, near Črna na Koroškem (1180 m asl; 46.45704 N, 14.93038 E). The plants were grown in experimental fields beginning on 28 May 2020 in Javorje and on 2 June 2020 in Ljubljana and Podbeže. Samples of plants were collected on 20 July 2020 in Podbeže, on 21 July in Javorje and on 22 July in Ljubljana, and biochemical measurements and anatomical analysis of leaves were performed in the laboratory. Samples were transferred from field to laboratory in polybags and kept at ambient temperature. In the experimental growth fields, we performed physiological measurements on plants between 27 July 2020 and 29 July 2020.

### 3.2. Biochemical Parameters

The content of photosynthetic pigments (chlorophyll a, b and carotenoids) was determined according to the method of Lichtenthaler and Buschmann (2001a and 2001b) [28,29]. Fresh leaf was collected, weighed and homogenized in a mortar, then extracted with acetone (90%). The homogenized samples were centrifuged for 4 min at 4000 rpm and 4 °C, and after centrifugation, the volume of the extract was determined. Extinction measurements using a VIS spectrophotometer (Lambda 12; PerkinElmer, Inc., Waltham, MA, USA) at 470, 645 and 662 nm were performed. The content of chlorophyll a and b and carotenoids per unit of dry weight of the sample was calculated using the measured extinction values.

The anthocyanin levels were measured using the method published by Lindoo and Caldwell in 1978 [30]. The dried lyophilized plant tissue was ground, covered and incubated with HCl:methanol:water (1:79:20 *v*/*v*/*v*) for 48 h in the dark at 3–5 °C. The homogenates were centrifuged for 4 min (4000 rpm and 4 °C), and then the absorbance at 530 nm of the extracts was measured using a UV/VIS spectrometer (Lambda 25; PerkinElmer, Inc., Waltham, MA, USA). After centrifugation, the volume of the extract was measured, and anthocyanin levels were expressed in relative units.

The content of UV-absorbing substances in buckwheat leaves was determined by the method described by Caldwell (1968) [31]. A powdered sample was extracted in extraction medium methanol:H_2_O:HCl = 79: 20: 1 (*v*/*v*/*v*). The sum of absorption values in the range 280–315 nm was used to calculate UV-B-absorbing compounds, and the sum of absorption values in the range 316–400 nm was used to calculate UV-A-absorbing compounds.

### 3.3. Physiological Parameters

The potential photochemical efficiency of PSII is expressed by Fv/Fm [Fv/Fm = (Fm − F0)/Fm] and was measured using a portable chlorophyll fluorometer (PAM 2500; Walz, Effeltrich, Germany). Fv is a variable fluorescence and F0 and Fm are the minimal and maximal chlorophyll fluorescence yields in dark adapted samples. For dark adaptation, samples were kept in cuvettes for 20 min before measurement. Fluorescence was excited with a saturating beam of “white light” (photosynthetic photon flux density, 8000 μmol/ m x s; 0.8 s). The effective quantum yield was determined by a saturating pulse of “white light” using a standard 60° angle clip, under saturating irradiance. Measurements of effective quantum yield were made at the prevailing ambient temperature. The effective quantum yield was defined as Y = (Fm’ – F0’)/Fm’; Fm’ is the maximum and F0’ the minimum fluorescence of an irradiated sample [32].

The degree of stomatal conductance was measured with a porometer (Decagon Devices, Inc., Pullman, WA, USA), which has a built-in thermometer to measure leaf temperatures. Measurements were performed on the same day and under the same conditions as the measurement of photochemical efficiency of FSII. We performed them on healthy developed leaves of plants (the youngest fully developed leaf) from 10 plants of each group of plants of the same species. Measurements were performed at each location three times in one day: at 9, 14 and 18 h. At the time of the measurements, the weather was clear.

### 3.4. Morphological Parameters

The numbers and dimensions of the CaOx druses in plants, the thickness of leaves, the thickness of palisade tissue and the thickness of epidermis were determined on transverse sections of leaves using light microscopy (CX41; Olympus, Japan) with a digital camera (XC30; Olympus, Japan) and the CellSens software (Olympus, Japan).

### 3.5. Statistical Analysis

Shapiro–Wilk tests were used to evaluate the normal distribution of the data. Homogeneity of variance from the means was assessed using Levene’s tests. Differences between treatments were tested using one-way analysis of variance followed by Tukey testing. To test for a statistically significant effect of factors (Location and Species) and interaction between both factors (Location x pecies), the factorial ANOVA was used. The level of significance was accepted at *p* < 0.05. The XLSTAT (version 2021.1.1, Addinsoft, 2022; New York, NY, USA) was used for these calculations.

## 4. Conclusions

Both species of buckwheat are well adapted to the conditions at different elevations. Tartary buckwheat did not respond to higher elevation with the production of UV-absorbing compounds, indicating better adaptation to high elevation conditions. Common buckwheat reacted to the climate conditions at the highest elevation with increased energy investment in secondary metabolism. According to our results, buckwheat responded differently to the conditions at different elevations. Deeper studies of metabolic profile, especially phenolics and proteins, will also enable us to find out the effects of different elevations upon the nutritional value of buckwheat.

## Figures and Tables

**Table 1:** Properties of soil and weather conditions of experimental locations Ljubljana, Podbeže and Javorje.

	Ljubljana	Podbeže	Javorje
Soil type	clay loam	loam–clay loam	loam–silt loam
Soil moisture (%)	17.5	15.4	14.7
EC (µS/cm)	80.8	75.9	180.2
C total (%)	2.8	3.1	8.4
N (%)	0.21	0.3	0.73
C org/N	12.4	10.0	11.4
Si (mg/g)	212	162	164
P (mg/g)	1.65	1.75	1.15
S (mg/g)	0.24	0.38	0.36
Cl (mg/g)	0.64	2.31	0.96
K (mg/g)	16.4	15.4	26.9
Ca (mg/g)	10.2	10.8	7.3
Average T June (°C)	19.4	17.4	9.7
Average T July (°C)	21.7	19.6	12.1
Rainfall June (mm)	142.7	189.8	170.9
Rainfall July (mm)	155.2	68.0	308.0

Legend: EC—electric conductivity; T—temperature, C total—total amount of carbon; C org—organic carbon.

**Table 2:** Contents of chlorophylls, anthocyanins and UV-absorbing compounds in common and Tartary buckwheat plants, grown at different elevations.

Model Parameter		Chlorophylls (mg/g d.w.)	Carotenoids (mg/g d.w.)	Anthocyanins (rel. u/g d.w.)	UV Abs. Comp. (rel u/g d.w.)
Location	Lj	17.2 ± 1.0 a	2.64 ± 0.14 a	0.18 ± 0.01 a	229 ± 11 ab
	Po	13.4 ± 0.7 b	2.42 ± 0.10 a	0.12 ± 0.01 b	217 ± 16 b
	Ja	15.6 ± 0.7 ab	2.57 ± 0.10 a	0.17 ± 0.01 a	277 ± 22 a
	Pr > F(Location)	**0.005**	0.306	**0.001**	**0.012**
Species	CB	15.4 ± 0.6 a	2.56 ± 0.08 a	0.15 ± 0.01	255 ± 15 a
	TB	15.4 ± 0.9 a	2.46 ± 0.10 a	0.16 ± 0.01	227 ± 15 a
	Pr > F(Species)	0.973	0.762	0.463	0.148
Location x Species	Pr > F(Location x Species)	**0.007**	**0.007**	0.382	**0.045**

Legends: CB—common buckwheat; TB—Tartary buckwheat; UV abs. comp.—absorbing compounds. Data are represented as means ± standard deviation. Means followed by different letters in the column are significantly different at *p* < 0.05 (n = 8). Statistical significances are marked with bold.

**Table 3:** Leaf thickness, palisade tissue thickness, epidermis thickness, number and diameter of CaOx druses.

Model Parameter		Leaf Thickness (μm)	Thickness of Palisade Tissue (μm)	Epidermis Thickness (μm)	Density of CaOx Druses (No/mm^2^)	2r of CaOx Druses (μm)
Location	Lj	156 ± 3 b	56 ± 1 b	24.1 ± 0.3 a	21.7 ± 0.7 a	18.2 ± 0.6 c
	Po	168 ± 5 a	66 ± 2 a	23.1 ± 0.4 a	17.3 ± 0.9 b	23.0 ± 0.6 a
	Ja	150 ± 4 b	51 ± 2 c	23.6 ± 0.5 a	17.4 ± 1.0 b	20.8 ± 1.3 b
	Pr > F (Location)	**0.0002**	**<0.0001**	0.112	**<0.0001**	**<0.0001**
Species	CB	168 ± 3 a	61 ± 2 a	24.3 ± 0.4 a	20.8 ± 0.6 a	23.0 ± 0.6 a
	TB	148 ± 3 b	54 ± 2 b	23.0 ± 0.3 b	16.8 ± 0.9 b	18.3 ± 0.7 b
	Pr > F (Species)	**<0.0001**	**0.0002**	**0.003**	**<0.0001**	**<0.0001**
Location x Species	Pr > F (Location x Species)	0.125	0.238	**0.007**	0.602	**<0.0001**

Legends: CB—common buckwheat, TB—Tartary buckwheat, Lj—Ljubljana, Po—Podbeže, Ja—Javorje. abs. comp.—absorbing compounds. Data are represented as means ± standard deviation. Means followed by different letters in the column are significantly different at *p* < 0.05 (n = 8). Statistical significances are marked with bold.

## Data Availability

Data are available from the authors on request.
